# Safety and effectiveness of immune checkpoint inhibitors in patients with preexisting autoimmune diseases: a systematic review

**DOI:** 10.3389/fimmu.2025.1712632

**Published:** 2025-11-18

**Authors:** Yasmim Dias, Viviane Silva, Maria Fernanda de Carvalho, Rafael Herchenhorn, Daniel Herchenhorn

**Affiliations:** 1 D’Or Institute for Research & Education (IDOR), Rio de Janeiro, Brazil; 2 Institute of Medical Education (IDOMED), University Estácio de Sá, Rio de Janeiro, Brazil

**Keywords:** autoimmune disease, immune checkpoint inhibitors, immune-related adverse events (irAEs), CTLA-4, PD-1, flares, safety, efficacy

## Abstract

**Background:**

Immune checkpoint inhibitors (ICIs) are effective in cancer treatment but may trigger immune-related adverse events (irAEs), especially in patients with preexisting autoimmune diseases (ADs). This population is often excluded from trials due to higher risks of flares, higher rates of irAEs, and potential reduced ICI efficacy. This review examines the safety and efficacy of immune checkpoint inhibitors (ICIs) in patients with preexisting autoimmune diseases and explores emerging evidence on potential predictive biomarkers.

**Methods:**

We conducted a systematic review using PubMed/MEDLINE, searching for articles published from 2015 to 2024 in English. The research combined terms for autoimmune diseases, ICIs (anti-CTLA-4, anti-PD-1/PD-L1), and cancer types, emphasizing studies reporting safety or efficacy outcomes. Due to marked heterogeneity in study design and outcomes, findings were summarized qualitatively rather than quantitative meta-analysis. The protocol followed PRISMA guidelines and was registered in PROSPERO (CRD420251037257).

**Results:**

We synthesized recent evidence from 17 studies including 883 cancer patients. with preexisting ADs treated with ICIs. The cohort predominantly included psoriasis (20.5%), rheumatoid arthritis (18%), and inflammatory bowel disease (17.2%) patients. Safety outcomes revealed that 53.5% experienced any-grade irAEs, including 27.5% with newly developed irAEs and 34.3% with autoimmune disease flares. Some patients experienced both irAEs and autoimmune flares concurrently, and 14.9% discontinued treatment due to toxicity (including 5 fatal cases, 0.5%). Treatment efficacy varied substantially, with overall response rates ranging from 11% to 50%, median PFS from 2.9 to 14.4 months, and median OS from 8.2 to 40.5 months. Significant heterogeneity in efficacy outcomes limited comparative analyses.

**Conclusions:**

These findings highlight that ICI therapy can be effective in selected patients with well-controlled autoimmune disease, but requires early monitoring, individualized treatment approaches, and multidisciplinary management of patients with coexisting autoimmune disorders.

## 1 Introduction

Immune checkpoint inhibitors (ICIs) have become a widely adopted cancer therapy since the FDA’s approval of the first anti-cytotoxic T-lymphocyte-associated protein 4 (CTLA-4) monoclonal antibody in 2011. CTLA-4 is a co-inhibitory receptor that dampens T-cell activity, primarily in lymph nodes. Its inhibition by drugs such as ipilimumab enhances the immune system’s ability to recognize and eliminate cancer cells. Similarly, inhibitors of programmed cell death protein 1 (PD-1) strengthen immune responses by preventing PD-1 from binding to its ligand, which normally suppresses tumor cell apoptosis and converts T effector cells into regulatory cells, impairing their cytotoxic function (1).

ICI therapy has demonstrated substantial efficacy and is generally associated with fewer cytotoxic side effects compared with conventional chemotherapy. However, blocking these immune checkpoints can also interfere with self-tolerance, potentially leading to immune-related adverse events (irAEs) that can affect any organ system at any point of treatment in a severe and unpredictable manner (2). The underlying mechanisms involve heightened activation of B and cytotoxic T cells, intracellular signaling dysregulation, and increased cytokine production, all of which contribute to inflammation (3–5). Recent rheumatology-focused studies further support this biological framework, showing that patients who develop rheumatic irAEs frequently display preexisting or induced autoantibody positivity, early B-cell activation, and persistent inflammatory pathways, which may extend beyond the treatment period. These findings provide mechanistic support for the early onset and variable severity of flares observed in patients with underlying autoimmune diseases (6, 7).

Autoimmune diseases (ADs) arise when the immune system mistakenly targets self-antigens, attacking the body’s own tissues. Given the increased risk of T-cell-mediated damage to healthy tissues and the close resemblance between ADs and irAEs, most patients with preexisting autoimmune conditions are typically excluded from early-phase and randomized prospective trials assessing ICI therapies (8, 9). However, with approximately 10% of cancer patients having preexisting ADs, it becomes crucial to better understand the efficacy, safety, and incidence of irAEs when ICIs are administered in cancer patients with concurrent ADs.

Although some ADs are better understood today, determining their prevalence and incidence remains difficult due to the lack of universal diagnostic criteria and standardized databases. Rising autoimmune disease prevalence causality remains multifactorial, reflecting improved diagnostic sensitivity, lifestyle patterns and environmental influences (e.g., obesity, infections). Some studies have shown an increased frequency of these diseases in recent decades, attributed not only to the world’s growing number of diagnoses but also to the rising prevalence of an important biomarker in ADs: antinuclear antibodies (ANA), which have been particularly studied in the USA (10, 11). Although ANA positivity has increased, it does not equate to clinical autoimmune disease in most cases, and its interpretation should be approached with caution.

These researches also indicate that Western and Northern countries, like the USA, have seen more recent cases compared to Southern and Eastern regions. Therefore, the role of Western lifestyle in the rise of ADs is being explored, with findings showing a positive association between obesity and lupus, sarcoidosis, and rheumatoid arthritis as well as a protective association between a diet rich in fruits, vegetables, and grains—which is less typical in the US—and these diseases (11–15).

Previous studies on the use of ICIs in patients with pre-existing ADs were primarily retrospective and uni institutional. Most of these studies were published during the early stages of ICI use, involving a variety of tumor types and ICI drugs. Because most clinical trials excluded patients with preexisting autoimmune disease, data on ICI safety and efficacy in this subgroup remain limited. Our review focuses on the relation between ICIs and pre-existing ADs, with particular emphasis on their impact on treatment efficacy, safety, and the identification of potential biomarkers to predict patient outcomes.

## 2 Methods

### 2.1 Study protocol

To address the research question, a systematic search of PubMed/MEDLINE was performed to identify studies evaluating the safety and efficacy of ICIs in cancer patients with pre-existing autoimmune diseases. The search strategy combined Medical Subject Headings (MeSH) and free-text terms related to autoimmune diseases, immune checkpoint inhibitors, and cancer types. A representative Boolean combination was: (“autoimmune disease” OR “rheumatoid arthritis” OR “psoriasis” OR “inflammatory bowel disease” OR “lupus”) AND (“immune checkpoint inhibitor” OR “PD-1” OR “PD-L1” OR “CTLA-4”) AND (“cancer” OR “neoplasm”). Retrospective and prospective studies with original data published between January 1, 2015, and September 1, 2024, were included. Duplicates were removed before screening. This time frame was chosen to reflect the period of widespread clinical implementation of immune checkpoint inhibitors and to exclude earlier case reports and small descriptive series that lacked systematic safety reporting. This review was conducted following PRISMA 2020 recommendations and was registered in PROSPERO (CRD420251037257).

### 2.2 Study selection

Specific inclusion and exclusion criteria were applied used to identify eligible articles for review. Eligible studies included patients with both cancer and autoimmune disorders receiving ICIs (anti-CTLA-4, anti-PD-1/PD-L1). The predefined primary outcomes included both safety and efficacy endpoints. Safety outcomes included autoimmune disease flares, newly developed immune-related adverse events (ND irAEs), total irAEs, fatal events, and treatment discontinuation due to toxicity. Efficacy outcomes comprised objective response rate, progression-free survival, and overall survival, according to study-defined or RECIST 1.1 criteria.

Studies were included if they reported at least one safety or one efficacy outcome, acknowledging the heterogeneity of reporting in real-world settings. This strategy was prespecified in the study protocol to maximize evidence capture while maintaining methodological transparency.

The review encompassed English-language studies published from 2015 to 2024, while excluding animal research, reviews without original data, studies lacking disease-specific outcomes, and non-English publications. The PRISMA flowchart of study selection can be found in **Supplementary Figure 1**. Quality assessment used the Newcastle–Ottawa Scale (selection, comparability, outcome domains); scores ranged 5–9, with two studies scoring 9, and most cohorts in the intermediate band (6–8). Comparability most often limited scores (few adjusted analyses/heterogeneous confounder control), with recurrent issues in outcome ascertainment and follow-up. Given this risk-of-bias profile and endpoint/reporting heterogeneity, we prespecified a qualitative synthesis and did not perform a meta-analysis. Individual NOS scores are presented in **Supplementary Table 1**. All but two of the included studies were retrospective in design; the two prospective studies (Loriot et al. and Danlos et al.) were observational and included small autoimmune disease subgroups, which limits their individual interpretability. Follow-up duration was extracted when explicitly reported, ranging from 4.7 to 22.8 months across studies. In several cohorts, follow-up was not available and is indicated as NA in **Supplementary Table 1**.

### 2.3 Data extraction and synthesis

Data extraction was performed using predefined parameters, including author, year of publication, country, study design, population characteristics (cancer type, autoimmune disease subtype, number of patients, disease status at treatment initiation), ICI regimen, and safety and efficacy outcomes. Missing data were recorded as not available without contacting authors, as specified in the protocol. Two independent reviewers (RH, VS) extracted data, and discrepancies were resolved through discussion and, when necessary, consultation with a third reviewer (YD) to ensure consistency in data collection. Inter-rater agreement was not formally quantified using statistics such as Cohen’s κ, which is acknowledged as a limitation of this review.

Given the substantial clinical and methodological heterogeneity across studies, including differences in tumor types, autoimmune disease subtypes, ICI regimens, and inconsistent definitions of immune-related adverse events, a formal meta-analysis was not appropriate. These differences, combined with incomplete reporting of key variables such as follow-up duration and denominators, precluded valid statistical pooling. In line with PRISMA and Cochrane guidance, which discourage pooled estimates when key assumptions of comparability are not met, we therefore opted for a structured qualitative synthesis. Safety outcomes were summarized qualitatively, and a descriptive subgroup analysis comparing CTLA-4 and PD-1/PD-L1 inhibitors is presented in **Supplementary Table 2**.

## 3 Results

**Figure 1:**
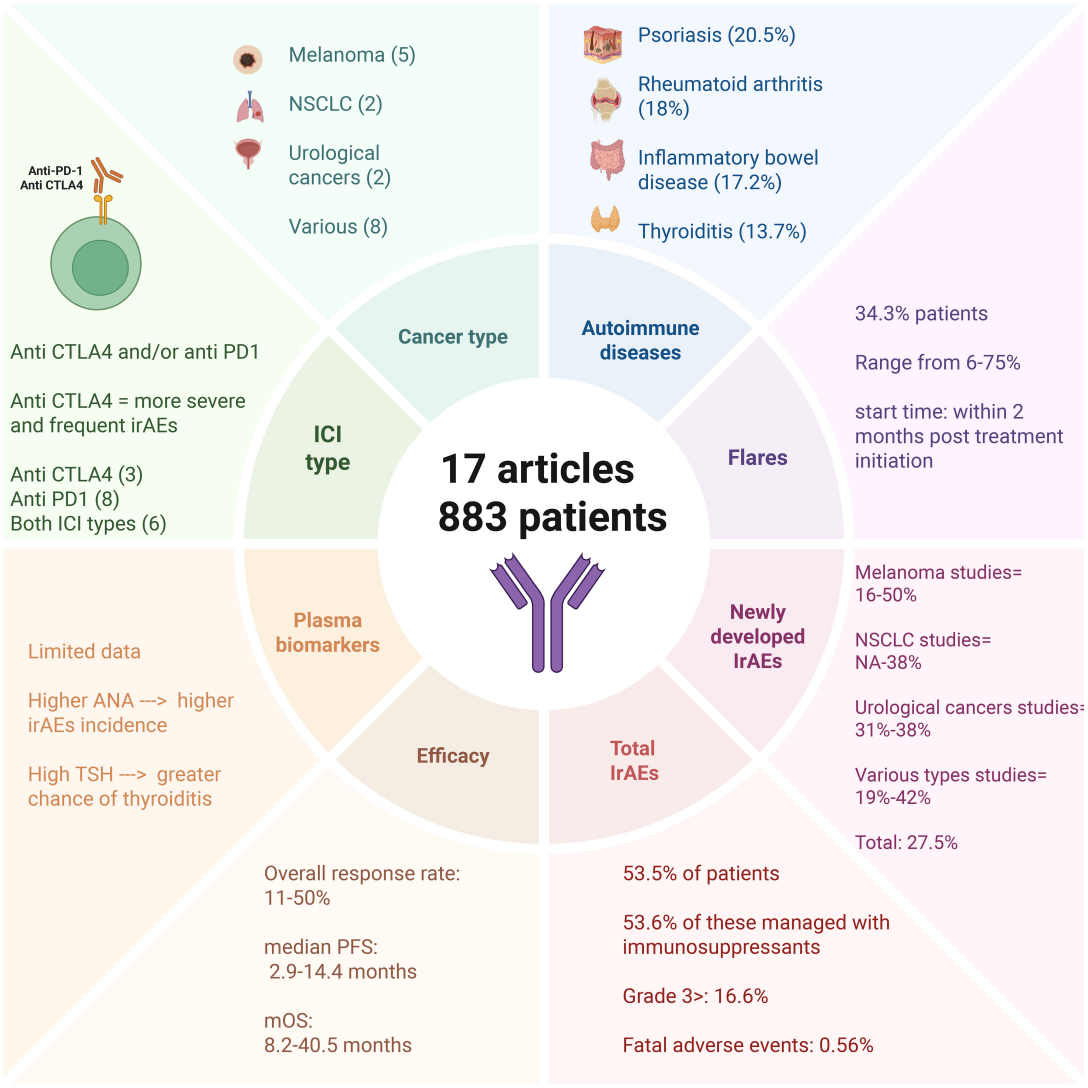
Concise overview of 17 studies (883 patients) of immune checkpoint inhibitors in patients with pre-existing autoimmune disease. Panels summarize cancer types and ICI classes (CTLA-4, PD-1/PD-L1), most represented autoimmune diseases, flares of baseline disease (often within 2 months of treatment start), newly developed irAEs, and aggregate safety/efficacy ranges (ORR, mPFS, mOS). Values reflect the original studies, may overlap, and are not additive due to heterogeneous reporting. Created with BioRender.com. Abbreviations: ORR, overall response rate; mPFS, median progression-free survival; mOS, median overall survival.

In total, 17 studies were included, assessing 883 cancer patients with pre-existing ADs who were treated with anti-CTLA-4 and/or anti-PD-1 immunotherapy—summarized in **Figure 1**. Of these, 9 studies evaluated patients with only one type of cancer: melanoma (n=5), NSCLC (n=2), and urological cancers (n=2). The remaining 8 studies evaluated multiple cancer types simultaneously. Regarding ADs, the most prevalent were psoriasis (20.5%), rheumatoid arthritis (18%), inflammatory bowel disease (17.2%), and thyroiditis (13.7%). Out of these patients, 280 had active diseases (31.7%) during ICI therapy.

Our analysis encompasses safety and efficacy outcomes of immunotherapies in these participants, as shown in **Tables 1** and **2**. The immune-related adverse events (irAEs) described in these studies were classified into two types. The first corresponds to the exacerbation of pre-existing ADs (flares), while the second includes newly developed (ND) irAEs, which do not have a clear causal relationship with pre-existing ADs. Both types are collectively referred to as total irAEs (TirAEs). Flares were observed in 303 patients (34.3%), and new-onset irAEs in 243 patients (27.5%). These events were not mutually exclusive, and some patients experienced both. However, because several studies did not provide disaggregated data, the exact number of overlapping cases could not be determined. These percentages therefore, reflect crude proportions and should not be interpreted as additive.

**Table 1:** Safety profile of immune checkpoint inhibitors in patients with preexisting ADs.

Cancer type	Target	Study	N	Active AD (%)*	Active AD (%)	Safety					
						Total irAEs (%)	irAEs grade ≥3 (%)	Preexisting AD flare (%)	Newly developed irAEs (%)	Use of systemic immunosuppressives (%)	Treatment discontinuation
Melanoma	CTLA-4	Kahler (16)	41	Thyroiditis (37%), Psoriasis (17%), RA (15%)	27	44	NA	29	29	NA	7 + 0
Melanoma	CTLA-4	Johnson (21)	30	RA (20%), IBD (20%), Psoriasis (17%)	43	50	33	27	33	43	NA
Melanoma	CTLA-4	Lee (22)	8	RA (100%)	NA	100	75	75	50	75	5
Melanoma	PD-1	Menzies (17)	52	RA (25%), Psoriasis (12%), Colitis (10%)	29	67%	15	38	29	53	2+4
Melanoma	PD-1	Gutzmer (23)	19	Thyroiditis (26%), RA (21%), Psoriasis (16%)	0	58	16	42	16	58	0
NSCLC	PD-1	Yoneshima (24)	18	ANA+ (100%)	0	33	11	NA	NA	5.6	2
NSCLC	PD-1	Leonardi (18)	56	Psoriasis (25%), RA (20%), Thyroiditis (16%)	18	55	14	23	38	20	0+8
Urol.	PD-1	Loriot (25)	35	Psoriasis (43%), Thyroiditis (17%), RA (11%)	31	46	14	11	31	37	3
Urol.	PD-1 /CTLA-4	Martinez (19)	106	Psoriasis (23%), Thyroiditis (13%), RA (11%)	33	73	18	36	38	37	6+8
Various	PD-1	Danlos (26)	45	Vitiligo (38%), Psoriasis (27%), Thyroiditis (16%)	55	44	11	24	20	13	5
Various	PD-1	Cortellini (27)	85	Psoriasis (40%), RA (27%), IBD (13%)	17	65	9	47	19	NA	6
Various	PD-1 /CTLA-4	Tison (28)	112	Psoriasis (28%), RA (18%), IBD (13%)	33	71	30	47	42	45	23
Various	PD-1 /CTLA-4	Richter (29)	16	RA (31%), PMR (31%), Sj\"ogren's (13%)	NA	38	25	6	31	38	6
Various	PD-1 /CTLA-4	Abu-Sbeih (30)	102	IBD (100%)	20	NA	20	41	NA	76	33
Various	PD-1 /CTLA-4	Braga (31)	13	IBD (100%)	30	NA	NA	31	NA	31	0
Various	PD-1 /CTLA-4	Efuni (20)	22	RA (100%)	14	73	9	55	32	83	2+1
Various	PD-1	Fountzilas (32)	123	Psoriasis (23%), RA (20%), DM1 (10%)	65	60	10	25	35	15	11

N refers to the total number of patients with pre-existing autoimmune disease (AD) included in each study. Flare, exacerbation of the underlying autoimmune disease; new irAE, newly developed immune-related adverse event. These outcomes are independent and not mutually exclusive. Combined rates are presented as reported in the original studies (Total irAEs column). Some patients experienced both events, but overlap was inconsistently reported. Discontinuation values are shown as two numbers separated by a plus sign (e.g., 2+4), indicating discontinuations due to flares and newly developed irAEs, respectively. When causes were not specified, discontinuation events were included in the TirAEs category. Percentages were calculated based only on studies reporting each endpoint; missing data were not imputed. NA, not available/applicable; AD, autoimmune disease; ANA, antinuclear antibody; DM1, type 1 diabetes mellitus; IBD, inflammatory bowel disease; irAE, immune-related adverse event; NSCLC, non-small cell lung cancer; PD-1, programmed death-1; PMR, polymyalgia rheumatica; RA, rheumatoid arthritis. NOS quality scores for all included studies are reported in **Supplementary Table 1**. *clinically active symptoms.

**Table 2:** Efficacy profile of immune checkpoint inhibitors in patients with preexisting ADs.

Cancer type	Target	Study	N	Efficacy		
				ORR (%)	mPFS (months)	mOS (months)
Melanoma	CTLA-4	Kahler (16)	41	12	NA	NA
Melanoma	CTLA-4	Johnson (21)	30	20	3.0	12.5
Melanoma	CTLA-4	Lee (22)	8	50	NA	NA
Melanoma	PD-1	Menzies (17)	52	33	6.2	NR
Melanoma	PD-1	Gutzmer (23)	19	32	NA	NA
NSCLC	PD-1	Yoneshima (24)	18	28	2.9	11.6
NSCLC	PD-1	Leonardi (18)	56	22	NA	NA
Urol.	PD-1	Loriot (25)	35	11	8.2	4.4
Urol.	PD-1/CTLA-4	Martinez Chanza (19)	106	35	NA	NA
Various	PD-1	Danlos (26)	45	38	NA	NA
Various	PD-1	Cortellini (27)	85	38	14.4	15.7
Various	PD-1/CTLA-4	Tison (28)	112	49	NA	NA
Various	PD-1/CTLA-4	Richter (29)	16	NA	NA	NA
Various	PD-1/CTLA-4	Abu-Sbeih (30)	102	NA	NA	NA
Various	PD-1/CTLA-4	Braga Neto (31)	13	NA	NA	NA
Various	PD-1/CTLA-4	Efuni (20)	22	NA	NA	10.5
Various	PD-1	Fountzilas (32)	123	NA	NA	40.5

N, total number of patients with pre-existing autoimmune disease (AD) included in each study. ORR, Overall Response Rate (complete + partial response, per study-defined criteria). mPFS, median Progression-Free Survival (treatment start to progression or death). mOS, median Overall Survival (treatment start to death from any cause). NR, Not Reached. NA, Not Available/Not Applicable (endpoint not reported for AD subgroup). Efficacy endpoints refer to the AD subcohort. No imputation was performed. Endpoints follow original study definitions (e.g., RECIST 1.1 for response, when applicable).

### 3.1 Flares

Flares, defined by acute exacerbations of preexisting autoimmune symptoms, occurred in 303 patients (34.3%). Most events developed early during ICI therapy, typically within the first two months. Exact timing varied across studies due to heterogeneous reporting and definitions; therefore, we did not pool estimates. This temporal pattern likely reflects early T-cell reactivation and accelerated immune activation following checkpoint blockade, which is consistent with the known kinetics of immune checkpoint inhibitors (1, 4). Clinically, flares often mirrored the underlying autoimmune disease, frequently affecting the same anatomical sites as previous episodes.

Regarding treatment discontinuation, five studies provided disaggregated data, allowing partial attribution of causes. In this subset, 17 discontinuation events (44.7%) were attributed to autoimmune flares and 21 (55.3%) to newly developed irAEs (16, 17–20). Across the full cohort, 132 patients (14.9%) discontinued treatment due to toxicity, including 5 fatal events (0.5%). However, in the majority of studies, discontinuation was reported only as TirAEs without further distinction. This prevented us from determining the relative contribution of flares and ND irAEs among all patients who discontinued therapy.

A limited number of studies examined baseline autoimmune disease activity as a potential modifier of flare risk. In Leonardi et al., flares occurred in 50% of patients with active disease compared to 18% with inactive disease (p = 0.04), with onset ranging from 1 to 260 days (18). Loriot et al. similarly reported that all flares occurred in patients with active disease at baseline, within the subgroup of 11 patients with active autoimmune disease, with timing from day 21 to 358 (25). In Menzies et al., flare rates were 60% in active versus 30% in inactive disease, with a median time to onset of 38 days (17). Martinez Chanza et al. reported flares in 40% of symptomatic versus 34% of asymptomatic patients, with a higher cumulative incidence at 3 months (30% vs 24%) (19). In the IBD cohort of Abu-Sbeih et al., active disease at baseline was associated with more severe gastrointestinal irAEs (median time to flare 62 days, IQR 33–123), though not with a higher overall incidence (30). Tison et al. reported heterogeneous flare frequencies across autoimmune subtypes, with flares in 40% of patients with rheumatoid arthritis and 59% of those with psoriasis; baseline activity was not uniformly predictive of flares across conditions (28). Efuni et al. described a median time to flare of one month in a rheumatoid arthritis cohort, although no activity stratification was provided (20).

These findings suggest that, in the subset of studies reporting this association, patients with clinically active disease at ICI initiation may have a higher and earlier risk of flares, particularly in rheumatologic and dermatologic conditions, although associations are not uniform and reporting remains incomplete and inconsistent across studies. Importantly, most included cohorts involved patients with mild to moderate autoimmune disease, and evidence remains sparse in patients with severe baseline activity.

### 3.2 Total IrAEs and newly developed IrAEs

Among the 883 patients analyzed, irAEs were reported in any grade in 472 (53.5%). Of these, 253 (53.6%) required immunosuppressants for management. newly developed adverse events occurred in 27.5% of cases (n=243), with 147 (16.6%) being grade 3 or higher. Reassuringly, only 5 patients (0.56%) had fatal outcomes, with most events occurring after a median of three treatment doses (16–32).

In the 5 melanoma-specific studies, total irAE rates varied widely (5%-100%), while new irAEs were observed in 16%-50% of cases (16–23). Kähler et al. noted that patients with previous flares were more likely to develop additional irAEs. Furthermore, alongside Lee et al. and Johnson et al., all three studies concluded that these adverse events were effectively managed using corticosteroid-based treatment algorithms (16, 21, 22). Menzies et al. demonstrated that 8% of treatment discontinuations were attributed to ND IrAEs (17).

For non-small cell lung cancer (NSCLC) studies, total irAE rates ranged from 33% to 55%, with new events in up to 38% of patients (18, 24). Leonardi et al. showed that 14% of patients permanently discontinued treatment due to ND irAEs, and the safety of immunotherapy in these patients was comparable to that of the general population, as the incidence of irAEs was similar to that observed in patients without ADs (18). In urological cancers, 46%-73% of patients developed irAEs, with 31%-38% being new developed events—mostly mild and reversible, particularly in asymptomatic cases. Only 8% discontinued therapy due to ND irAEs (19, 25).

Across eight studies with heterogeneous cohorts (various tumor types), total irAE incidence ranged from 38% to 73%, with new events in 19%-42% of cases (20, 26–32). Danlos et al. demonstrated significantly shorter irAE-free survival in AD patients (5.4 vs. 13 months) (26), while discontinuation rates varied from 7% to 38% (20, 26–32).

As with many previous analyses, Fountzilas et al. and Efuni et al. also concluded that most adverse events were not severe, concluding that immunotherapy remains safe for this population when accompanied by close monitoring and prompt intervention for significant toxicity (20, 32).

### 3.3 Efficacy

The efficacy of ICIs in patients with pre-existing autoimmune diseases may be influenced by factors such as prior corticosteroid or antibiotic use (4, 33), but available evidence suggests these patients can still achieve meaningful clinical benefit. Only a subset of studies reported objective response rates (ORR, n=12), progression-free survival (PFS, n=5), and median overall survival (mOS, n=6). Because of heterogeneity in patient populations and cancer types, direct comparisons across studies were not performed.

Reported ORRs ranged from 11% to 50% (16–32). In advanced melanoma cohorts, response rates generally exceeded 30% (Menzies et al.; Gutzmer et al.), consistent with outcomes observed in non-AD populations (17, 23). Median OS ranged from 8.2 to 40.5 months (16–32), with the lowest survival reported by Loriot et al. in advanced urothelial carcinoma (8.2 months), with no significant difference compared to patients without autoimmune disease (8.8 months) (25). Reported OS and PFS were within ranges observed in non-AD populations, although formal statistical comparisons were not possible.

While these findings are derived from heterogeneous and limited datasets, they indicate that meaningful clinical benefit is possible in this population, but should be interpreted with caution.

## 4 Discussion

The use of ICIs in patients with pre-existing ADs presents unique clinical challenges. A central concern involves balancing treatment efficacy against the risk of autoimmune flares and irAEs, with specific risks varying by underlying AD type. Additionally, the choice between anti-CTLA-4 and anti-PD-1/PD-L1 agents may significantly influence toxicity profile. The impact of corticosteroids on ICI efficacy remains controversial. Some studies show comparable outcomes, while others report reduced response rates and shorter progression-free survival.

Emerging research has identified several promising biomarkers, including plasma markers, genetic signatures, and microbiota profiles, that may help predict toxicity risk (34–39). Similarly, new approaches like selective immunosuppression and microbiome modulation show promise for irAE management, although they lack validation for routine use in practice (38, 39). This discussion examines these critical considerations through analysis of both our review data and existing literature, providing insights for managing this complex patient population.

### 4.1 AD-specific risks

When focusing specifically on patients with preexisting IBD, Abu-Sbeih et al. studied 102 IBD patients (49 Crohn’s disease, 49 ulcerative colitis) receiving immunotherapy and reported gastrointestinal adverse events in 41% of cases, with a median onset of 62 days, including 21 cases (20.6%) of grade 3–4 diarrhea (30). Similarly, Braga Neto et al.’s analysis of 13 IBD patients (5 Crohn’s, 8 ulcerative colitis) identified flares in 4 patients (30.7%), with median onset at 5 months post-ICI initiation and requiring corticosteroid treatment. In our broader cohort, which included various autoimmune conditions, 34.3% experienced flares (30). Consistent with Abu-Sbeih’s findings, most flares in our cohort occurred early, primarily within the first two months of ICI therapy. IBD flares mirror those in other autoimmune conditions. Like other irAEs, they typically occur early in treatment. This pattern emphasizes the importance of close initial monitoring.

When focusing specifically on patients with rheumatoid arthritis (RA), the findings from Efuni and Lee highlight possible differences when compared to the broader autoimmune population included in our study (20, 22). Efuni et al. evaluated 22 RA patients treated with ICIs, reporting a flare rate of 54.5% (12 patients), with most flares occurring around one month after therapy initiation. Of those, 10 responded well to corticosteroid treatment, reaffirming that most flares are well managed with steroids, as mentioned previously. Additionally, 32% of patients experienced irAEs, 9% of which were grade 3 (20). Lee et al. smaller series (N = 8) showed higher rates of 75% irAE incidence and 62.5% grade ≥ 3 events, though corticosteroids also proved effective in management. By comparison, our larger cohort (N = 883) demonstrated 27.2% new irAE incidence and 33.4% flare rate (22). This suggests RA patients may experience both higher flare frequencies and greater severe irAE burdens than general autoimmune populations, though Lee’s small sample size (N = 8) could lead to potential selection bias.

### 4.2 ICI-specific risks: anti CTLA4 x anti PD1/PDL1

Among the 17 included studies, 11 specifically evaluated CTLA-4 (3) or PD-1/PD-L1 inhibitors (8), while the remaining studies included mixed cohorts. Among the studies reporting toxicity data, the overall incidence of irAEs was similar between CTLA-4 (51.9%) and PD-1/PD-L1 inhibitors (58.2%). However, the proportion of grade ≥3 irAEs was markedly higher in CTLA-4 recipients (42.1% vs 11.8%), as were treatment discontinuations due to toxicity (24.5% vs 9.4%). Autoimmune flares (32.9% vs 30.6%) and newly developed irAEs (32.9% vs 28.4%) were slightly more frequent in the CTLA-4 group, though these differences were modest. Denominators varied according to reporting availability, and missing data was not imputed. Given reporting heterogeneity and incomplete outcome reporting, no formal statistical comparison was performed (16–18, 21–27, 32).

These findings are presented in **Supplementary Table 2**, which summarizes the comparative safety profile of both drug classes. While the overall incidence of irAEs was comparable, the higher proportion of severe events and discontinuations associated with CTLA-4 blockade suggests a more clinically significant toxicity burden, with potential implications for patient selection and treatment course.

Mechanistically, anti–PD-1/PD-L1 agents more commonly induce thyroiditis and pneumonitis, whereas anti–CTLA-4 therapies are particularly associated with hypophysitis and colitis. This gastrointestinal predilection is especially relevant for patients with preexisting inflammatory bowel disease (IBD). In a study of 102 IBD patients (17% treated with ipilimumab), anti-CTLA4 therapy showed a non-significant but clinically relevant trend toward increased gastrointestinal toxicity in multivariate analysis (p=0.58), while demonstrating significance in univariate analysis (30). This likely reflects the study’s limited ipilimumab cohort (n=17). Supporting evidence comes from a smaller study (n=13) reporting substantially higher colitis incidence with anti-CTLA4 versus PD1 inhibitors (9.1% vs 1.6%) (31). These findings emphasize the need for thorough risk-benefit evaluation before administering anti-CTLA4 agents to IBD patients with close monitoring to ensure early intervention.

### 4.3 Use of corticosteroids/immunosuppressives

The impact of concomitant corticosteroid use on ICI efficacy remains unclear. Kähler and colleagues conducted a retrospective analysis on the safety and efficacy of ipilimumab in 41 patients with advanced melanoma and pre-existing AD (16). Of these, 11 patients (27%) received immunosuppressive therapy at baseline. Despite the small sample size, the authors found comparable ORR between immunosuppressed (9%) and non-immunosuppressed patients (13.3%) (16). Consistent with this finding, Gutzmer and Leonardi et al. reported no significant impact of immunosuppressive therapy on ICI efficacy (18, 23).

In contrast, the study by Menzies et al. included 20 patients (38%) on immunosuppressants at the start of treatment—including corticosteroids (17%), steroid-sparing agents (SSAs, 10%), or both (10%)—and showed a significantly lower response rate in the immunosuppressed subgroup (3/20, 15%) compared to non-immunosuppressed patients (14/32, 44%) (p = 0.033) (17). They also noted a trend toward more frequent flares in immunosuppressed patients (50% vs. 31%), although this did not reach statistical significance (p > 0.05) (17). These observations were supported by Martinez Chanza and Kähler et al., who also reported increased AD exacerbations in immunosuppressed patients (16, 19).

Similarly, Fountzilas et al. demonstrated that baseline corticosteroid use correlated with shorter PFS (HR = 2.08, 95% CI 1.18–3.68, p = 0.012), while other immunomodulators showed no such effect (32). Corroborating these findings, Tison et al. reported that patients receiving immunosuppressive drugs at the start of ICI therapy had shorter PFS (3.8 vs. 12 months, p = 0.006), although there was no difference in OS (28). In their study, irAEs/flares were associated with worse PFS (HR = 1.97, 95% CI 1.06–3.66, p = 0.032) but not OS, likely due to the statistical design. Multivariate analysis revealed the worst outcomes in patients who discontinued treatment or required immunosuppressants for irAE/flare management (28).

Notably, in broader ICI-treated populations, high-dose baseline corticosteroids (≥10 mg prednisone equivalent) have been consistently associated with reduced response rates and survival, providing a clinically relevant threshold to interpret these findings (40–42).

Data on background immunosuppressants are mixed and likely depend on dose and timing. In patients with ADs, baseline corticosteroid use has been associated with reduced PFS, consistent with evidence from larger ICI cohorts identifying high-dose exposure (≥10 mg prednisone equivalent) as a predictor of poorer outcomes. In contrast, short-course corticosteroids introduced after ICI initiation for irAE management do not appear to compromise efficacy (41, 42).

### 4.4 Biomarkers

The identification of robust biomarkers is crucial for personalizing immune checkpoint inhibitor (ICI) therapy in patients with pre-existing autoimmune diseases. Although several candidate markers have been investigated, our analysis did not demonstrate significant associations between most biomarkers and the development of irAEs, underscoring the complexity of their role. It is therefore important to clarify how these exploratory biomarkers may be applied, whether to support risk stratification, guide prognostic assessment, or inform future therapeutic strategies. In this context, we structured the discussion into three categories: (1) autoantibodies (ANA and anti-Tg) as candidate risk markers, (2) genomic alterations as research-stage predictors, and (3) microbiome as an experimental but promising therapeutic target.

#### 4.4.1 Autoantibodies (ANA, anti-Tg)

Autoantibodies have been the most frequently explored biomarkers in this setting. In a retrospective study by Yoneshima et al. evaluating anti–PD-1 therapy in NSCLC, 21% of patients were ANA-positive (24). Although overall irAE rates were similar between ANA-positive (33.3%) and ANA-negative (32.3%) patients, a trend toward increased incidence was observed at higher ANA titers. ANA positivity was also associated with shorter median overall survival (11.6 vs. 15.8 months; p < 0.05), suggesting a possible negative prognostic signal (24).

Beyond ANA, elevated baseline thyroid-stimulating hormone (TSH) and anti-thyroglobulin antibodies (anti-Tg) have been associated with an increased risk of autoimmune thyroid dysfunction during ICI therapy (34, 35, 43, 44). These markers may help identify patients at higher risk for specific irAEs, though their predictive value remains exploratory.

#### 4.4.2 Genomic mutation

Genomic alterations represent another class of exploratory biomarkers that may help identify patients at increased risk of immune-related toxicities. In this context, Wells et al. investigated the association between somatic mutations and the development of irAEs in 60 patients with metastatic melanoma treated with anti–CTLA-4 or anti–PD-1 therapy (37). Among those who developed irAEs (30% of the cohort), mutations were identified in immunoregulatory genes such as *CTLA4*, *PD1*, *JAK1*, and *HLA*, as well as in inflammatory mediators including *TNF* and *IL6*. Notably, tumor mutational burden (TMB) alone was not predictive, suggesting that the presence of specific gene alterations, rather than overall mutation load, may contribute to susceptibility to autoimmunity (36, 37, 43). These observations highlight a promising but still early research avenue that requires validation in larger and more diverse patient populations.

#### 4.4.3 Microbiome

The gut microbiome has also emerged as a promising exploratory biomarker with potential relevance for both risk assessment and therapeutic modulation. Chaput et al. conducted a groundbreaking study identifying specific bacterial signatures associated with colitis risk, particularly enrichment of Bacteroidetes (such as Bacteroides fragilis) and reduced microbial diversity (37). These findings have been translated into experimental therapies, with case reports demonstrating complete colitis resolution following fecal microbiota transplantation in refractory cases (38). However, significant limitations remain, including high interindividual variability, confounding by medications such as antibiotics and proton pump inhibitors (PPIs), and the lack of standardized sampling and analysis protocols. As with other biomarker classes, microbiome-based strategies remain investigational and require prospective validation before being incorporated into clinical practice.

### 4.5 Therapeutic approaches

A personalized approach to managing ADs in patients receiving immunotherapy, balancing the risk of worsening AD without compromising treatment efficacy, is the primary goal of treatment. Unfortunately, most current specific approaches, such as microbiome transplantation and the use of selective immunosuppressors (SIs), come from small retrospective studies or are in early stages, with limited clinical validation.

A potential approach for managing irAEs in patients with ADs is the two-step therapeutic approach. In the first step, referred to as the rotation phase, non-selective immunosuppressors (nSIs) such as corticosteroids (CS), mycophenolate mofetil (MMF), azathioprine (AZA), methotrexate (MTX), and cyclophosphamide (CYC) are proposed to be discontinued and replaced with selective immunosuppressors (SIs), including tocilizumab (IL-6 receptor antibody), infliximab (anti-TNF-α antibody), and vedolizumab (α4β7 integrin antibody). ICIs would then be initiated after 2–4 weeks of stable disease under SI therapy. In cases of rapidly progressive disease, where timely treatment is critical, ICI initiation may occur concurrently with SIs. In the second step, called the maintenance phase, the concomitant use of ICIs and SIs is proposed to mitigate the risk of severe irAEs and AD flares (39). This two-step approach remains hypothetical and requires validation in prospective studies before routine adoption. However, it offers valuable insights to guide future studies.

### 4.6 Limitations

This systematic review has several limitations that must be considered when interpreting the findings. First, there was substantial heterogeneity across the included studies regarding cancer types (melanoma, NSCLC, urological cancers), pre-existing autoimmune diseases (psoriasis, rheumatoid arthritis, inflammatory bowel disease and others), and treatment regimens (anti-CTLA-4, anti-PD1/PDL1, monotherapy vs. combination therapy). All but two of the included studies were retrospective in design, reflecting the scarcity of prospective evidence in this population. The two prospective studies (Loriot et al. and Danlos et al.) were observational and included small autoimmune disease subgroups, which limits their standalone interpretability. Most studies were small, with 10 enrolling fewer than 50 patients. This variability, along with heterogeneity in reporting, precluded formal meta-analysis, and subgroup analyses were underpowered.

Additionally, the lack of standardized definitions and reporting methods for irAEs and autoimmune disease flares may have introduced measurement bias. The absence of a formal risk-of-bias assessment using ROBINS-I also represents a methodological limitation that should be considered when interpreting the results. Regarding biomarkers, while antinuclear antibodies (ANA) and gut microbiome signatures show preliminary predictive value for irAEs, the absence of prospective validation studies and standardized criteria currently prevents their clinical implementation.

Evidence largely derives from Western, tertiary-care cohorts, potentially underrepresenting patients with severe or non-Caucasian autoimmune phenotypes, which further limits the generalizability of the findings. These limitations highlight the need for cautious interpretation of our findings and underscore the importance of future prospective, controlled studies to validate these results and guide personalized therapeutic approaches for this complex patient population.

## 5 Conclusion

This systematic review explores both the potential and challenges of using ICIs in patients with cancer and ADs. While ICIs have demonstrated significant clinical benefits, their use is accompanied by a notably higher risk of immune-mediated complications, including autoimmune flares and *de novo* irAEs. In the reviewed studies, approximately half of patients experienced these events, a finding that supports the need for cautious patient selection, individualized treatment planning, and multidisciplinary monitoring rather than suggesting universal exclusion from ICI therapy. In appropriately selected patients with well-controlled autoimmune disease, ICIs may be administered with close surveillance, allowing clinical benefit while mitigating risk.

With the broader use of ICIs, future progress will require prospective registries, data coming from real-world studies, validated risk stratification models, and standardized steroid-tapering protocols to improve patient selection, minimize toxicity, and optimize clinical outcomes.

## Supplementary material

Supplementary Figure 1 – PRISMA flowchart of study selection.
