# Supplementary material for: Safety and effectiveness of immune checkpoint inhibitors in patients with preexisting autoimmune diseases: a systematic review
Source: Front Immunol. 2025 Nov 18;16:1712632. doi: 10.3389/fimmu.2025.1712632 (PMC12669149; doi:10.3389/fimmu.2025.1712632)
Supplement: Supplementary Figure 1 — – PRISMA flowchart of study selection. [file Supplementaryfile1.zip › Supplementary Table 2.DOCX]

## Supplementary Table 2. Comparative safety profile of CTLA-4 and PD-1/PD-L1 inhibitors in patients with cancer and pre-existing autoimmune disease

| Group | N total | Total irAEs (%) | irAEs grade ≥3 (%) | Preexisting AD flare (%) | Newly developed irAEs (%) | Treatment discontinuation (%) |
| --- | --- | --- | --- | --- | --- | --- |
| CTLA-4  [16-18] | 79 | 41/79  (51.9%) | 16/38  (42.1%) | 26/79  (32.9%) | 26/79  (32.9%) | 12/49  (24.5%) |
| PD-1/PD-L1  [19-23, 25-26, 32]. | 433 | 252/433  (58.2%) | 51/433  (11.8%) | 127/415  (30.6%) | 118/415  (28.4%) | 41/433  (9.4%) |

1. **Supplementary Table 2**. Comparative safety of anti–CTLA-4 versus anti–PD-1/PD-L1 in patients with cancer and pre-existing autoimmune diseases.
2. Denominators vary by endpoint and include only patients from studies reporting that outcome; missing data were not imputed (e.g., flare and ND-irAEs were not reported in Yoneshima; treatment discontinuation was not reported in Johnson).
3. Flares and ND-irAEs are not mutually exclusive; percentages are descriptive and not intended for formal statistical comparison.
4. Abbreviations: AD, autoimmune disease; AD flare, acute exacerbation of the underlying autoimmune disease; CTCAE, Common Terminology Criteria for Adverse Events; CTLA-4, cytotoxic T-lymphocyte–associated protein 4; PD-1, programmed cell death protein 1; PD-L1, programmed death-ligand 1; irAE, immune-related adverse event; ND-irAE, newly developed immune-related adverse event; Total irAEs, composite of AD flares and ND-irAEs (events are not mutually exclusive); Treatment discontinuation, discontinuation of ICI due to toxicity, when reported; NA, not available.
