# Supplementary figures and images for: Safety and effectiveness of immune checkpoint inhibitors in patients with preexisting autoimmune diseases: a systematic review
Source: Front Immunol. 2025 Nov 18;16:1712632. doi: 10.3389/fimmu.2025.1712632 (PMC12669149; doi:10.3389/fimmu.2025.1712632)

# **Appendix 1**

**Supplementary figure 1 – PRISMA flowchart of study selection**


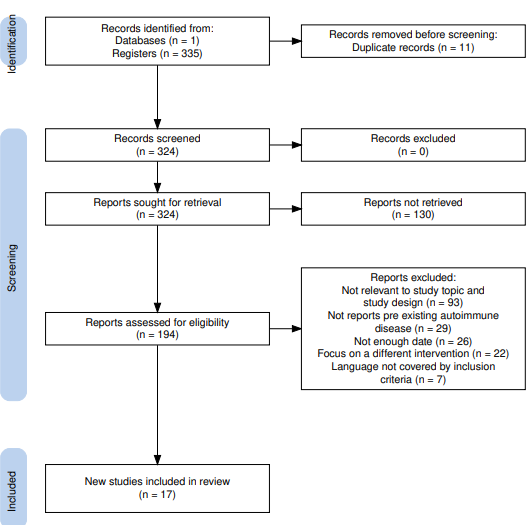

Supplement: Supplementary file 2 [file DataSheet1.docx]
